# Gray Matter Volume of a Region in the Thalamic Pulvinar Is Specifically Associated with Novelty Seeking

**DOI:** 10.3389/fpsyg.2018.00203

**Published:** 2018-02-27

**Authors:** Daphne Stam, Yun-An Huang, Jan Van den Stock

**Affiliations:** ^1^Laboratory for Translational Neuropsychiatry, Department of Neuroscience, KU Leuven, Leuven, Belgium; ^2^Old Age Psychiatry, University Psychiatric Center KU Leuven, Leuven, Belgium; ^3^Brain and Emotion Laboratory, Maastricht University, Maastricht, Netherlands

**Keywords:** novelty seeking, voxel-based morphometry, thalamus, pulvinar, impulsiveness

## Abstract

Personality reflects the set of psychological traits and mechanisms characteristic for an individual. Geno-neuro-biologically inspired personality accounts have proposed a set of temperaments and characters that jointly compose personality profiles. The present study addresses the link between neurobiology and personality and investigates the association between temperament traits and regional gray matter volume. Furthermore, the specificity of these associations as well as the underlying components that drive the association are addressed. One hundred and four participants completed the Temperament and Character Inventory (TCI) and underwent structural magnetic resonance brain imaging. The participants included premanifest carriers of Huntington's disease, as this population is associated with temperament-related neuropsychiatric symptoms. Whole brain voxel-based multiple regression analyses on gray matter volume revealed a significant specific positive correlation between a region in the left thalamic pulvinar and novelty seeking score, controlled for the other traits (P_height_ < 0.05, FWE-corrected). No significant associations were observed for the other temperament traits. Region of interest analyses showed that this association is driven by the subscale NS2: impulsiveness. The results increase the knowledge of the structural neurobiology of personality and indicate that individual differences in novelty seeking reflect the structural differences observed in the brain in an area that is widely and densely connected, which is in line with the typically domain-general behavioral influence of personality traits on a wide range of affective, perceptual, mnemotic, executive, and other cognitive functions.

## Introduction

Some people love the thrill of waterskiing while others start panicking at the very idea. Personality reflects the set of psychological traits and mechanisms characteristic for an individual (Larsen and Buss, 2010). In the psychobiological personality account of Cloninger et al. (1993), personality is conceptualized as composed of temperaments and characters. Temperaments are regarded to be stable and consistent over time and are involved in behaviors linked with emotions or arousal. They emerge early in life and are presumed to have a heritable basis (Cloninger, 1986; Cloninger et al., 1993; Larsen and Buss, 2010).

Four temperaments are typically considered: (1) novelty seeking (NS) reflects enthusiasm, quick-temperedness, impulsivity, and reward-sensitivity; (2) harm avoidance (HA) is related to acting with caution and passive avoidance behavior; (3) reward dependence (RD) is associated with responsiveness to signals of reward; and finally persistence (P) indicates motivation without direct external reward (Cloninger, 1987; Cloninger et al., 1993; Uzman, 2003; Larsen and Buss, 2010; Laricchiuta et al., 2014).

Character traits, on the other hand develop as an interaction between genetic factors and experiences (learning, sociocultural factors), which change throughout the course of life.

Three character scales have been proposed: (1) self-directedness is related to responsibility and purposefulness; (2) cooperativeness is linked to helpfulness and empathy; (3) self-transcendence reflects self-forgetfulness and believe in spiritual acceptance (Cloninger, 1987; Cloninger et al., 1993; Uzman, 2003; Larsen and Buss, 2010).

Temperaments are posited to be heritable and homogeneous, stable over time, and independent of each other (Cloninger, 1986; Cloninger et al., 1993; Heath et al., 1994; Stallings et al., 1996; Comings et al., 2000; Uzman, 2003).

The present study investigates the structural neurobiology associated with temperaments. There is general consensus that behavioral and psychological phenoma have a brain correlate and hence that inter-individual differences in behavior and personality are related to inter-individual brain differences. Indeed, temperaments are related to neurobiological variables at the molecular level across a wide range of species. For instance, NS is associated with endocannabinoid, dopamine and glutamate receptor availability in humans (Zald et al., 2008; Van Laere et al., 2009; Leurquin-Sterk et al., 2017) and rodents (Häring et al., 2011; Parkitna et al., 2013).

In the present study, we focus on associations between temperaments and regional gray matter volume. Several studies have previously investigated this association. Considering the domain-general impact of personality traits, it could be hypothesized that the associations are located in areas that are widely and densely connected throughout the brain, e.g., rich club hubs (van den Heuvel and Sporns, 2011). The results have been largely inconsistent so far. For instance, novelty seeking (NS) has been associated with gray matter (GM) volume of the left middle frontal gyrus (Iidaka et al., 2006), the right superior and middle frontal gyrus and posterior cingulate gyrus (Gardini et al., 2009), and the cerebellum (Picerni et al., 2013; Laricchiuta et al., 2014). Although two studies found an association between NS and the middle frontal gyrus (Iidaka et al., 2006; Gardini et al., 2009), the respective clusters do not overlap. In fact they are in separate hemispheres and separated about 5 cm along the anterior-posterior axis. These inconsistencies may be due to methodological issues. See Table 1 for a schematic overview of the methods and results of previous studies.

**Table 1:** Overview of studies investigating GM associations with temperament traits of the TCI/TPQ; novelty seeking (NS), harm avoidance (HA), reword dependence (RD), and persistence (P).

**Reference**	**#Participants**	**Focus - Resolution**	**Covariates**	**Statistical threshold**	**Results**	**R/L**	**#Voxels**	***Z*-value**	**Coordinates**		
								**(*t-*value)**	**x**	**y**	**z**
Iidaka et al., 2006	65 [30 males]	Whole brain – voxel level	Sex, age, BDI score, total GM volume	*p* = 0.001 (*k* = 100)	**Novelty seeking:**						
					MFG^a^	L	525	4.21	−48	41	35
					**Harm avoidance:**						
					Amygdala^b^	L	136	3.31	−17	−1	−18
					Orbital gyrus	L	355	3.85	−12	61	−24
					MTG	R	122	3.51	74	−35	6
					Angular gyrus	R	142	3.39	51	58	60
					**Reward dependence:**						
					Caudate nucleus^c^	R	224	3.72	27	−31	11
					ITG	R	443	3.55	71	−30	−22
					ITG	R	727	3.55	52	−37	−20
					STG	R	182	3.45	47	24	−32
Gardini et al., 2009	85 [58 males]	Whole brain -	Sex, age, education	*p* < 0.05	**Novelty seeking:**						
		voxel level		(FDR-corrected)	SFG/MFG	R	1,292	3.29	12	14	54
					SFG/PCG	R	1,135	3.06	10	35	49
					**Harm avoidance:**						
					Precuneus/MOG	L	3,941	4.84	−22	−75	34
					IFG/MFG	L	2,645	4.22	−52	23	22
					IFG	R	1,528	4.16	55	30	−11
					Cuneus/IPL	R	776	3.93	32	−81	34
					**Reward dependence:**						
					Caudate nucleus/	R	10	2.82	14	8	12
					Rectal gyrus						
					**Persistence:**						
					Precuneus	L	23	2.78	0	−73	51
					Paracentral lobule/	R	22	2.67	12	−36	62
					Parahippocampal gyrus						
Picerni et al., 2013	100 [43 males]	Cerebellum – voxel level	Sex, age, education	*P* < 0.05	**Novelty seeking:**						
				(FWE-corrected) (k = 10)	Lobule VIII	L	359	5.33	−26	−51	59
Laricchiuta et al., 2014^d^	125 [52 males]	Cerebellum – cortical hemisphere level	Sex, age, total brain volume	*P* < 0.01	**Novelty seeking:**						
					Cerebellar cortex	L		(3.46)^e^			
					Cerebellar cortex	R		(2.97)^f^			
					**Harm avoidance:**			0			
					Cerebellar cortex	L		(−2.16)^g^			
					Cerebellar cortex	R		(−1.76)^h^			

*Coordinates refer to MNI-space. Signed Differential Mapping was used to convert Talairach coordinates to MNI coordinates (https://www.sdmproject.com/utilities/?show=Coordinates). MFG, Middle frontal gyrus; MTG, Middle temporal gyrus; ITG, Inferior temporal gyrus; STG, Superior temporal gyrus; SFG, Superior frontal gyrus; PCG, Posterior cingulate gyrus; MOG, Middle occipital gyrus; IFG, Inferior frontal gyrus; IPL, Inferior parietal lobule; WM, white matter*.

a *Left prefrontal gray matter survived SVC (p = 0.04)*.

b *Left amygdala region survived SVC (p = 0.01)*.

c *Right caudate nucleus survived SVC (p = 0.008)*.

d *The described results were published in the supporting information, we included these results as they are controlled for age, sex, and total brain volume*.

e *Degrees of freedom: (114)*.

f *Degrees of freedom: (115)*.

g *Degrees of freedom: (113)*.

h *Degrees of freedom: (114)*.

In the present study, we combine voxel-based (VBM) with surface-based morphometry (SBM) and stringent statistical procedures to investigate the association between temperaments and regional gray matter volume and cortical thickness. Furthermore, 2 additional issues are addressed that may increase the knowledge of the structural neurobiology of personality: (1) the specificity of temperament-brain associations and (2) temperament characteristics driving the temperament-brain association.

To investigate the former, we included all four traits (NS, HA, RD, and P) in a single statistical regression model. In previous studies a correlation among traits was taken as argumentation to perform separate regression analyses for each scale (Gardini et al., 2009). This procedure results in reduced specificity of the results. Including all four traits in a single model on the other hand maximizes the specificity of the results of a single trait (as it controls for the association that is contained by the other traits). To investigate the second additional issue, i.e., the characteristics driving a trait-brain association, every significant trait-brain association is followed up by an analysis investigating the association between GM and the trait subscales. The temperaments can be divided into different subscales: NS consists of four subscales: exploratory excitability (NS1), impulsiveness (NS2), extravagance (NS3), and disorderliness (NS4); HA can be divided into anticipatory worry (HA1), fear of uncertainty (HA2), shyness (HA3), and fatigability (HA4); RD is linked to sentimentality (RD1), social attachment (RD2), and dependency (RD3); the temperament trait P is not divided into different subscales (Cloninger et al., 1993).

## Methods

This study was carried out in accordance with the recommendations of Ethical Committee of University Hospitals Leuven. All subjects gave written informed consent in accordance with the Declaration of Helsinki.

### Participants

One hundred and four healthy subjects participated [33 males (33 %); mean age ± *SD* = 35 ± 11 years, range 18–75; 2 lefthanded]. The sample was composed of three subgroups to increase the variability of the loadings on personality scales: (1) Twenty participants with premanifest Huntington's disease (19% [45% male]), (2) Nineteen gene-negative controls from Huntington's disease families (18% [37% male]), (3) 65 healthy controls (63% [27% male]).

We included participants with premanifest Huntington's disease, as Huntington's disease is associated with TCI-temperament-related neuropsychiatric symptoms, e.g., impulsivity and apathy (Rosenblatt, 2007) and neuropsychiatric symptoms are frequently observed in the premanifest stage of Huntington's disease (Martinez-Horta et al., 2016).

### Temperament and character inventory

The Dutch version of the 240-item (true-false) TCI was used. The Dutch version of the TCI is a validated translation and the temperament dimensions of the Dutch version have reasonable to good psychometric internal consistency (Cronbach's α range, 0.64–0.87) and include a validation in a representative sample of Dutch individuals (*n* = 1034) (version 1.3; Datec Psychological Tests, Leiderdorp, the Netherlands). Representative items of the TCI include: “I like to explore new ways to do things (NS),” “I often feel tense and worried in unfamiliar situations, even when others feel there is little to worry about (HA),” “I like to please other people as much as I can (RD)” and “I am more of a perfectionist than most people (P).”

### Brain imaging

#### MRI acquisition

A high-resolution T1-weighted anatomical image (voxel size: 0.98 × 0.98 × 1.20 mm^3^) was acquired on a single 3T Philips Achieva system equipped with a 32 channel head coil using a 3D turbo field echo sequence (TR:9.6 ms; TE:4.6 ms; matrix size:256 × 256; 182 slices).

#### Structural data analysis

Data was analyzed using CAT12, a Computational Anatomy Toolbox (http://www.neuro.uni-jena.de/cat/) (Gaser and Kurth, 2017) running under SPM12 (http://www.fil.ion.ucl.ac.uk/spm/software/spm12/) and MATLAB (R2016b). To investigate associations between temperaments and regional GM volume, we performed voxel-based morphometry (VBM). Preprocessing consisted of normalization to MNI space, tissue classification (segmentation) into GM, white matter (WM), and cerebrospinal fluid (CSF), and bias correction of intensity non-uniformities (default parameter settings). The amount of volume changes due to spatial registration were scaled, in order to retain the original local volumes (modulating the segmentations). The modulated images were smoothed using a 12 × 12 × 12 mm full-width at half-maximum Gaussian kernel. In order to investigate associations between temperaments and cortical thickness we performed surface-based-morphometry (SBA). Preprocessing included normalization to MNI space, local adaptive segmentation, cortical thickness and central surface estimation, topological correction, spherical mapping, and spherical registration. Projection-based thickness was used to estimate cortical thickness and create the central cortical surface for the left and right hemisphere. The modulated images were smoothed using a 15 × 15 × 15 mm full-width at half-maximum Gaussian kernel.

#### Statistical analysis

Statistical tests on behavioral variables were preceded by a normality check on the distributions of the respective residuals by means of Shapiro-Wilk test. In case normality could not be assumed, non-parametric tests were performed.

A multiple regression analysis was performed on the smoothed GM-images of the entire sample. The four temperament scores (NS, HA, RD, and P) were entered as regressors in a single model, in addition to age, sex, and total intracranial volume (TIV), which were included as variables of no interest. The statistical threshold was set at P_height_ < 0.05, corrected for familywise error (FWE).

Two similar multiple regression analysis were performed on the smoothed cortical thickness images, one for the left and one for the right hemisphere. The multiple regression model was similar to the one on the GM-images, except for the inclusion of TIV as a covariate.

To identify which subscales drive any temperament-brain associations, we performed ROI analyses. We conducted a multiple regression analysis with all the subscales (of the ROI-defining main scale) as regressors and age, sex and TIV as regressors of no interest. The statistical threshold was set at P_height_ < 0.001, combined with FWE correction at cluster level.

Anatomic labeling of significant clusters was performed in xjView (http://www.alivelearn.net/xjview) and clusters were visualized using MRICron (http://www.mccauslandcenter.sc.edu/mricro/mricron).

## Results

We first investigated group differences in the TCI-scores. Shapiro-Wilk test showed that residuals of NS, HA, and RD were normally distributed (*P* > 0.097), P was not normally distributed (*P* = 0.001). Kruskal–Wallis tests showed no significant group effect on the NS, HA, RD, and P score (*P* > 0.345).

**Figure 1:**
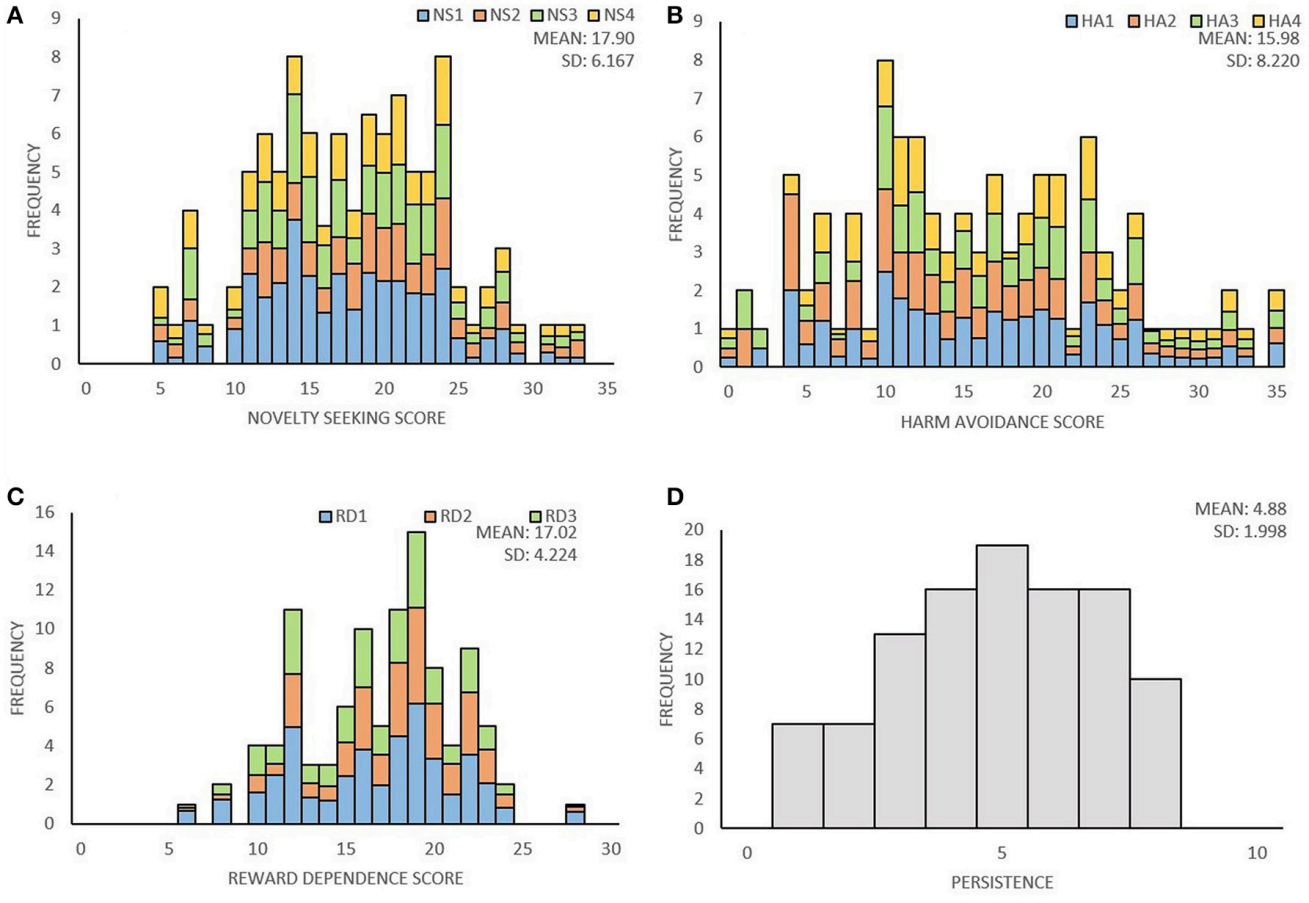
Distribution of the four temperament traits of the TCI. **(A)** Distribution of novelty seeking score and the percentage for each subscale; exploratory excitability (NS1), impulsiveness (NS2), extravagance (NS3), and disorderliness (NS4). **(B)** Distribution of harm avoidance score and the percentage for each subscale; anticipatory worry (HA1), fear of uncertainty (HA2), shyness (HA3), fatigability (HA4). **(C)** Distribution of reward dependence score and the percentage for each subscale; sentimentality (RD1), social attachment (RD2), dependency (RD3). **(D)** Distribution of persistence score.

Distribution of temperament traits in the present sample and the mean and standard deviation (SD) of the temperament scales are shown in Figure 1. Subsequently, we investigated associations between the main- and subscales by computing partial correlation coefficients (controlling for age and sex) between the four main scales; novelty seeking (NS), harm avoidance (HA), reward dependence (RD), persistence (P), and their subscales. The results are presented in Table 2. Partial correlations between the TCI main scales showed that there were no high correlations (< I0.431I) between the four traits. In addition, we ran a multicollinearity check which showed no problematic collinearity (variance inflation factors (VIF) < 1.3).

**Table 2:** Partial correlation between the four main scales; novelty seeking (NS), harm avoidance (HA), reward dependence (RD), persistence (P), and their subscales.

	**NS**	**HA**	**RD**	**P**	
NS	–	−0.431^**^	0.207^*^	−0.128	
HA	−0.431^**^	–	0.009	−0.165	
RD	0.207^*^	0.009	–	−0.088	
P	−0.128	−0.165	−0.088	–	
	**NS**	**NS1**	**NS2**	**NS3**	**NS4**
NS	–	0.551^**^	0.652^**^	0.715^**^	0.640^**^
NS1	0.551^**^	–	−0.006	0.335^**^	0.151
NS2	0.652^**^	−0.006	–	0.294^**^	0.357^**^
NS3	0.715^**^	0.335^**^	0.294^**^	–	0.239^*^
NS4	0.640^**^	0.151	0.357^**^	0.239^*^	–
	**HA**	**HA1**	**HA2**	**HA3**	**HA4**
HA	–	0.869^**^	0.799^**^	0.826^**^	0.750^**^
HA1	0.869^**^	–	0.712^**^	0.658^**^	0.544^**^
HA2	0.799^**^	0.712^**^	–	0.583^**^	0.442^**^
HA3	0.826^**^	0.658^**^	0.583^**^	–	0.457^**^
HA4	0.750^**^	0.544^*^	0.442^**^	0.457^**^	–
	**RD**	**RD1**	**RD2**	**RD3**	
RD	–	0.672^**^	0.804^**^	0.466^**^	
RD1	0.672^**^	–	0.253^*^	0.070	
RD2	0.804^**^	0.243^*^	–	0.220^*^	
RD3	0.466^**^	0.070	0.220^*^	–	

* P < 0.05;

** *P < 0.001*.

### Correlation between temperaments and voxel-wise GM volume

**Figure 2:**
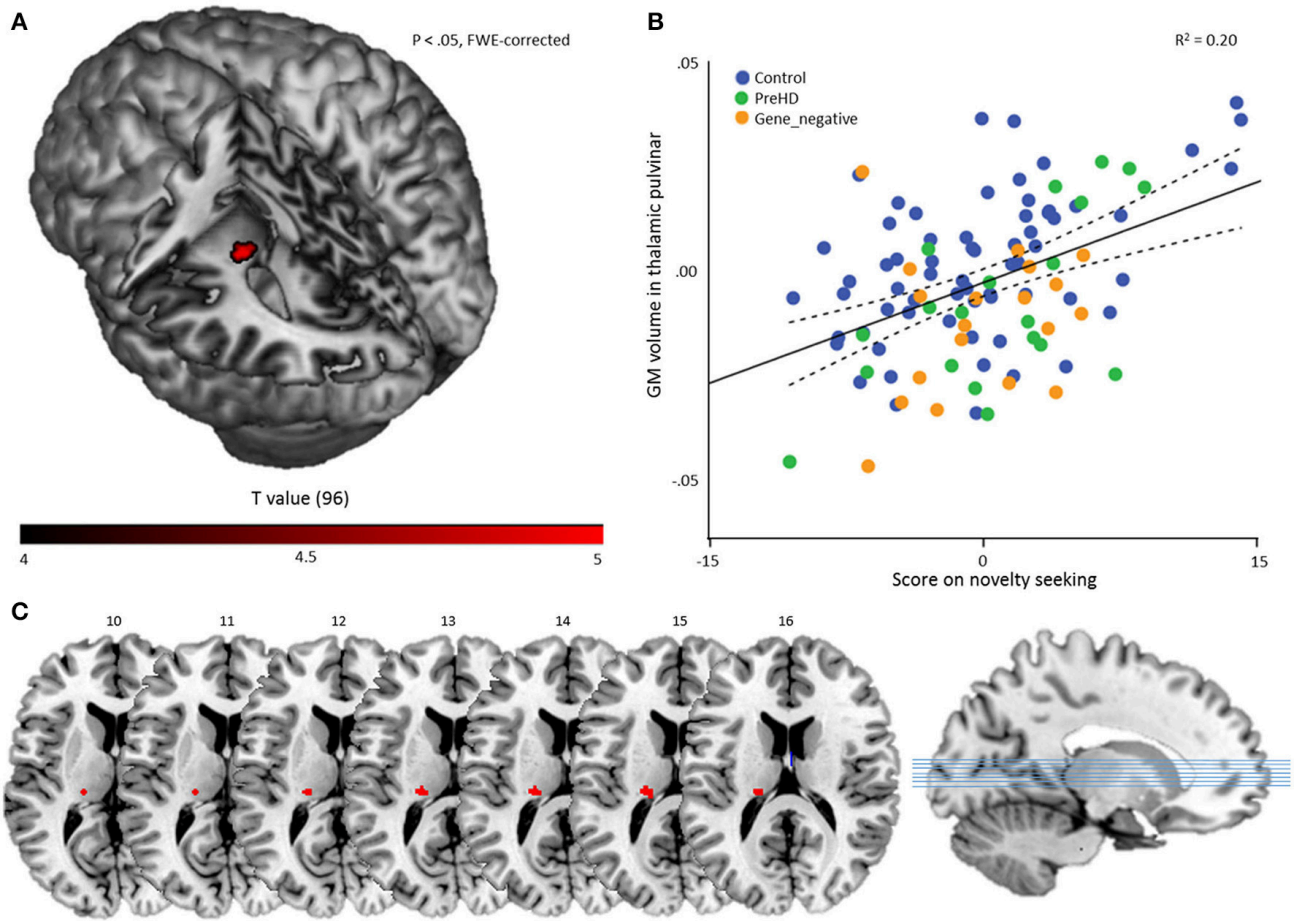
Multiple regression results. **(A)** A statistical map displaying the association between novelty seeking score and GM volume of a region in the left thalamic pulvinar. The result is overlaid on a canonical 3-dimensional–rendered MRI brain template with a cut-out (left). **(B)** Scatterplot showing the partial correlation (*r* = 0.444) between the mean GM volume in the thalamic pulvinar as a function of novelty seeking, controlled for age, sex, TIV, HA, RD, and P. Color coding refers to group membership. The solid line refers to the fitted linear regression and the dashed lines refer to the mean confidence interval. **(C)** Cluster on axial slices, with the numbers referring to MNI Z-coordinates.

To investigate temperament-specific associations with GM volume, we ran a multiple regression analysis on the smoothed GM images with NS, HA, RD, P, age, sex, and TIV as covariates. Eight contrasts were performed: for every temperament scale, we investigated the positive and negative association (P_height_ < 0.05, FWE-corrected). This revealed only one significant result: a positive association between NS and a cluster in the left thalamic pulvinar (60 voxels, peak voxel: *x* = −20, *y* = −27, *z* = 14; *t* = 5.03; *P*_height_ = 0.011, FWE-corrected; *Z* = 4.73), Figure 2.

This cluster remained significant in a post-hoc multiple regression analysis with NS, age, sex, and TIV as covariates (and hence without HA, RD, and P) (*t* = 3.17; *P*_height_ < 0.001, combined with SVC *P*_height_ = 0.003, FWE-corrected at cluster level).

### ROI-analysis

To investigate whether the association was disproportionally driven by (a) subscale(s), we performed a multiple regression analysis in the ROI on the smoothed GM images with NS1, NS2, NS3, NS4, age, sex, and TIV as covariates. Four contrasts were performed, evaluating the positive correlation for every subscale. This revealed a significant result for the NS2 (impulsivity) scale (*t* = 3.18; *P*_height_ < 0.001, combined with SVC *P*_height_ = 0.004, FWE-corrected at cluster level; peak voxel characteristics: *X* = −23; *Y* = −24; *Z* = 14; *t* = 3.48; pFWE = 0.002; *Z* = 3.37).

As a secondary analysis, we computed for every subject the mean volume in the ROI and performed a partial correlation analysis with NS score, controlling for age, sex, TIV, HA, RD, and P. This revealed, as expected, a significant result: *r* = 0.444 (*P*_height_ < 0.001) and indicates that the volume of the cluster explains about 20% of the NS variance in the sample (Figure 2).

### Correlation between temperament traits and cortical thickness

To investigate temperament-specific associations with cortical thickness, we performed 8 contrasts following the multiple regression analysis on the smoothed cortical thickness images with NS, HA, RD, P, age, and sex as covariates. For every temperament scale, we investigated the positive and negative association. This analysis was performed in both hemispheres (*P*_height_ < 0.05, FWE-corrected) and revealed no significant results.

## Discussion

In the current study, we investigated voxel-wise GM volume and cortical thickness associations with temperaments. We found a specific significant positive correlation between a cluster located in the left pulvinar nucleus of the thalamus and NS score. As a rich club hub, the thalamus is widely and densely connected throughout the brain (van den Heuvel and Sporns, 2011). This is in line with its structural association with a personality trait as the behavioral impact of personality traits is typically domain-general and influences a wide range of affective, perceptual, mnemotic, executive, and other cognitive functions. Individuals that score high with respect to NS, tend to be enthusiastic, quick-tempered, impulsive (Uzman, 2003; Larsen and Buss, 2010) and sensitive to reward (Cloninger, 1987). Furthermore, people that score high on NS tend to show intense excitement in response to novelty. This can lead to high emotional sensations and experiences (Cloninger, 1987; Cloninger et al., 1993).

The thalamic pulvinar is the largest nucleus in the primate brain (Halassa and Kastner, 2017). It is a region that is able to gate information from and control the primary visual cortex (V1) (Bender, 1981; Benevento and Miller, 1981; Purushothaman et al., 2012; Tamietto and Morrone, 2016) and also has multiple connectivity with other areas of the visual system (including regions in occipital, temporal, parietal, and frontal cortex; Van den Stock et al., 2014; Bridge et al., 2016; Halassa and Kastner, 2017). These multiple connections with the thalamic pulvinar suggest that NS may influence visual processing, possibly related to novelty detection (Lawson et al., 2012), which is associated with novelty seeking. Besides connections with visual cortical areas the thalamic pulvinar also shows connections with other sensory-specific cortical areas, like the auditory and the premotor cortex (Budinger et al., 2006; Cappe et al., 2009a,b; Tyll et al., 2011). In addition to a strong intensity dependence of visual signals in individuals that score high on sensation seeking, this has also been reported for auditory characteristics (Zuckerman and Neeb, 1979; Juckel et al., 1995). Furthermore, it has been suggested that the inter-individual variability in response to hearing music and music preference may be influenced by temperament traits (Gerra et al., 1998; Nater et al., 2005). NS had also been associated with speeded motor responses (risk-taking) (Forstmann et al., 2008; Hu et al., 2016). In line with this, an influence through preconceptual bias in perceptual memory and habit formation is a proposed mechanism of temperaments (Cloninger et al., 1993).

An important aspect of NS is emotion. Individuals that score high with respect to NS, tend to be enthusiastic and quick-tempered (Uzman, 2003; Larsen and Buss, 2010). A recent study showed that NS score is positively correlated with heightened emotion expression of joy (Giner-Bartolomé et al., 2016). We previously reported that increased irritability in premanifest carriers of the Huntington mutation is associated with increased activation in the pulvinar during experience of anger (Van den Stock et al., 2015). Similarly, structural and functional deterioration of the pulvinar plays a central role in behavioral variant frontotemporal dementia, phenotypically characterized by both hyperirritability and apathy (Lee et al., 2014).

Several reports have also implicated the pulvinar in detection of and orientation to emotional stimuli, both at the conscious (de Gelder et al., 2004) and unconscious level (de Gelder and Hadjikhani, 2006; Tamietto and de Gelder, 2010; Van den Stock et al., 2011), as well as at the behavioral response level (Ward et al., 2005), possibly through connectivity with the amygdala (Tamietto et al., 2012). This was confirmed by a study that suggested that the pulvinar plays a direct role in fear recognition, and an indirect role through cortical connections with the amygdala (Ward et al., 2005, 2007) and a recent study suggesting that emotional valence of visual stimuli is processed via subcortical pathway from the superior colliculus that reaches the amygdala via the pulvinar (Rafal et al., 2015). Furthermore, a recent review shows common activation in the amygdala and pulvinar in response to non-consciously processed emotional signals (Diano et al., 2017).

Our findings contrast with previous results reporting associations between NS on the one hand and cortical (frontal and posterior cingulate) and cerebellar volumes on the other hand (Iidaka et al., 2006; Gardini et al., 2009; Laricchiuta et al., 2014). These inconsistencies may be due to differences in methodology, e.g., a focus on the whole brain or on regions of interest (Table 1). Furthermore, we studied the specificity of temperament-brain associations, by including all four traits (NS, HA, RD, P) in a single statistical regression model combined with the strictest corrective method (FWE corrected). Including all four traits in a single model maximizes the specificity of the results of a single trait (as it controls for the association that is contained by the other traits).

Also in contrast with previous studies, we did not observe clusters that showed a significant correlation with one of the other temperament traits (HA, RD, P). A possible explanation is the conservative statistical threshold that we used in the present study, in order to reduce type I errors, in combination with the larger and more heterogeneous sample, which may reduce the chance of false positive results.

## Limitations and future directions

NS has been linked to several neuropsychiatric disorders, including drug addiction (Bardo et al., 1996; Lin et al., 2015; Vanhille et al., 2015), alcohol and tobacco abuse (Palmer et al., 2013), pathological gambling (Kim and Grant, 2001), eating disorders (Krug et al., 2009), post-traumatic stress disorder (PTSD) (Jakšić et al., 2012), and depression (Duclot and Kabbaj, 2013), yet the association with GM volume of the pulvinar remains to be investigated.

We previously reported that cerebral mGluR5 receptor availability is associated with temperament traits in healthy humans, with pulvinar mGluR5 availability explaining up to 50% of the NS variance in the sample (Leurquin-Sterk et al., 2016). It would be interesting to investigate whether the GM volume and mGluR5 receptor availability are associated in the pulvinar and how this relates to NS.

Some limitations are of note. First, the sex-ratio of our sample was unbalanced, as 72% was female. However, there was no difference in NS score between males and females and we statistically controlled for sex. Second, age was not normally distributed, with most participants in the range 20–40. To control for this, age was entered as variable of no interest in all analyses. Furthermore, as the study is correlational in nature, any causal interpretations are unjustified.

In conclusion, the present findings reveal a specific association between NS and GM volume of the pulvinar. This association is specifically driven by the impulsivity dimension of NS. These findings add to our understanding of the neurobiology of personality and may provide cues for its breakdown in psychopathology.

## Author contributions

DS and JV: contributed conception and design of the study and organized the database. DS: performed the statistical analysis and wrote the first draft of the manuscript. Y-AH wrote a script to calculate mean GM volume in the ROI for each subject. All authors contributed to manuscript revision, read and approved the submitted version.

### Conflict of interest statement

The authors declare that the research was conducted in the absence of any commercial or financial relationships that could be construed as a potential conflict of interest. The reviewer, AC, and handling Editor declared their shared affiliation.
